# Editorial to “Impact of intracardiac pattern matching settings on the activation map of accessory pathways using open window mapping”

**DOI:** 10.1002/joa3.70050

**Published:** 2025-03-25

**Authors:** Yosuke Nakatani

**Affiliations:** ^1^ Division of Non‐Pharmacological Management of Cardiac Arrhythmias Gunma University Graduate School of Medicine Maebashi Gunma Japan

In the case report published in the Journal of Arrhythmia,^1^ Morioku et al. evaluated the contribution of the intracardiac pattern matching (ICPM) algorithm, integrated within the CARTO3 system (Biosense Webster Inc., Diamond Bar, CA, USA), to the accuracy of annotations in open window mapping (OWM) for atrioventricular accessory pathway (AP). They compared the frequency of misannotations under various settings: without ICPM, based solely on atrial potentials, based solely on ventricular potentials, and based on both atrial and ventricular potentials. The results showed that ICPM reduced the frequency of misannotations, particularly when both atrial and ventricular potentials were utilized, demonstrating the greatest reduction in the misannotations (frequency of misannotations: without ICPM 0.75% vs. ICPM based on atrial and ventricular potentials 0.1%).

Recent advancements in 3D mapping systems have enabled high‐density mapping, significantly enhancing the mapping accuracy. High‐density mapping addresses the challenges of localizing accessory pathways using conventional methods, which involve point‐by‐point mapping to identify the connection sites of the AP to the atria or ventricle, or the site where the AP potential is recorded. The accuracy of 3D mapping improves with an increase in the mapping points. However, manually annotating local electrograms with a large number of mapping points can be challenging. Therefore, automatic annotation is necessary for high‐density mapping, but a misannotation to the incorrect chamber could compromise the mapping accuracy. The issue of misannotations is particularly crucial in mapping atrioventricular APs as it necessitates mapping near the valvular annulus, where misannotations of far‐field potentials from the ventricle during atrial mapping or from the atria during ventricular mapping can occur. To address this problem, OWM, which sets a mapping window that includes both atrial and ventricular potentials, was devised.^2^

Two critical premises must be met to guarantee the accuracy of the OWM. The first is that the near‐field potential is correctly annotated. OWM generally uses the dV/dt value in the unipolar signal to annotate near‐field potentials. To enhance the accuracy of those annotations, it is essential to eliminate the influence of far‐field potentials on the recorded potentials. A dedicated mapping catheter with a small electrode size and short inter‐electrode distance can be useful in eliminating the influence of far‐field potentials.^3^ We have reported that a multispline mapping catheter with closely spaced microelectrodes (OCTARAY, Biosense Webster) is beneficial for OWM.^4^

The second premise is that OWM is performed during the correct rhythm. Ectopic beats are frequently induced by mechanical stimulation during contact mapping, leading to misannotations. However, this problem has not been sufficiently solved so far. The ICPM algorithm automatically identifies changes in unipolar signals recorded by the reference catheter and assigns each pattern to its respective map. Using this method, only potentials recorded during the target rhythm are reflected in the map, potentially reducing misannotations due to ectopic beats during OWM. Therefore, Morioku et al.^1^ suggested that ICPM could fill a missing piece in ensuring the accuracy of OWM. Another important point in their report is that by utilizing both atrial and ventricular potentials for ICPM, the efficacy of the ICPM is further enhanced. The use of “open window ICPM” for OWM is particularly interesting, considering the similarity of the principles of both methods.

OWM is already a sophisticated method, yet Morioku et al.^1^ are striving to enhance it further by incorporating additional technology. Recent technological advancements have automated many mapping processes, diminishing the opportunities for us to exhibit our creativity. Nonetheless, the capability to effectively utilize these advancements in diagnostics and treatments still relies on our innovative ideas. I am grateful to the authors for enlightening me on this matter.

## FUNDING INFORMATION

None.

## CONFLICT OF INTEREST STATEMENT

Dr. Nakatani is affiliated with an Endowed Department funded by DVx Inc., Medtronic Japan, Biotronik Japan, Boston Scientific Japan, and Japan Lifeline.
